# Water Arsenic Exposure and Intellectual Function in 6-Year-Old Children in Araihazar, Bangladesh

**DOI:** 10.1289/ehp.9501

**Published:** 2006-10-18

**Authors:** Gail A. Wasserman, Xinhua Liu, Faruque Parvez, Habibul Ahsan, Pam Factor-Litvak, Jennie Kline, Alexander van Geen, Vesna Slavkovich, Nancy J. LoIacono, Diane Levy, Zhongqi Cheng, Joseph H. Graziano

**Affiliations:** 1 Department of Psychiatry, College of Physicians and Surgeons, Columbia University, New York, New York, USA; 2 New York State Psychiatric Institute, New York, New York, USA; 3 Columbia University Mailman School of Public Health, New York, NY, USA; 4 Lamont-Doherty Earth Observatory, Columbia University, Palisades, New York, USA

**Keywords:** arsenic, assessment, Bangladesh, children, water

## Abstract

**Background:**

We recently reported results of a cross-sectional investigation of intellectual function in 10-year-olds in Bangladesh, who had been exposed to arsenic from drinking water in their home wells.

**Objectives:**

We present results of a similar investigation of 301 randomly selected 6-year-olds whose parents participated in our ongoing prospective study of the health effects of As exposure in 12,000 residents of Araihazar, Bangladesh.

**Methods:**

Water As and manganese concentrations of tube wells at each home were obtained by surveying all study region wells. Children and mothers were first visited at home, where the quality of home stimulation was measured, and then seen in our field clinic, where children received a medical examination wherein weight, height, and head circumference were assessed. We assessed children’s intellectual function using subtests drawn from the Wechsler Preschool and Primary Scale of Intelligence, version III, by summing weighted items across domains to create Verbal, Performance, Processing Speed, and Full-Scale raw scores. Children provided urine specimens for measuring urinary As and were asked to provide blood samples for blood lead measurements.

**Results:**

Exposure to As from drinking water was associated with reduced intellectual function before and after adjusting for water Mn, for blood lead levels, and for sociodemographic features known to contribute to intellectual function. With covariate adjustment, water As remained significantly negatively associated with both Performance and Processing Speed raw scores; associations were less strong than in our previously studied 10-year-olds.

**Conclusion:**

This second cross-sectional study of As exposure expands our concerns about As neurotoxicity to a younger age group.

We recently reported an adverse association between drinking water arsenic and cognitive function in 10-year-old children living in Bangladesh (Wasserman et al. 2004), where approximately 30–40 million people (British Geological Survey and Bangladesh Department of Public Health Engineering 2001) are chronically exposed to naturally elevated levels of arsenic in groundwater pumped from approximately half the total of approximately 10 million tube wells in the country. That finding supplements reports of neurologic sequelae of acute and chronic exposure in adults (Bolla-Wilson and Bleecker 1987; Morton and Caron 2003; Morton and Dunnette 1994; Pershagen et al. 1981; Schoolmeester and White 1980). Two other studies have examined children’s intellectual function, in a small sample of children from a Mexican lead smelter area (Calderon et al. 2001) and from Taiwanese children in regions with and without elevated As in well water (Tsai et al. 2003). Both reported negative associations with As exposure, although the domains of vulnerable intellectual function differed across investigations.

In a related investigation (Wasserman et al. 2006), we recently observed poorer intellectual functioning, primarily in visual motor skills, in 10-year-olds from a region in Bangladesh where household well water concentrations were variable in manganese but extremely low in As. These findings were consistent with other investigations of motor function in children exposed to high levels of Mn (e.g., Takser et al. 2003).

We sought to expand these findings on children’s intellectual function to a younger age group, drawing once again on children of adults participating in our prospective study of the health effects of As in Araihazar, Bangladesh. Wells in the study site, a 25-km^2^ region located approximately 30 km east of Dhaka, are characterized by a wide range of As concentrations in drinking water, with 75% exceeding the World Health Organization (WHO) As standard of 10 μg/L, and 53% exceeding the Bangladesh standard of 50 μg/L (van Geen et al. 2003a). We report here the results of a cross-sectional investigation of intellectual function in 301 6-year-old children.

## Methods

### Overview

The current project is part of a larger ongoing multidisciplinary study by health, earth, and social scientists working collaboratively in Araihazar, Bangladesh. Earlier we described the region and the larger cohort study from which the present investigation is derived (Wasserman et al. 2004). As in most of rural Bangladesh, people in Araihazar live in houses with cement or mud floors and tin or straw walls and roofs. Members of extended families live in clusters of individual houses (a *bari*), surrounded by family farmland. Each bari has one or more tube wells, usually owned by a senior family member. This region is not particularly poor by Bangladeshi standards. Informed oral parental consent and child assent were obtained before the onset of the study, which was approved by the Bangladesh Medical Research Council and the Columbia University Institutional Review Board.

### Subjects

Of the 11,749 adults enrolled in our cohort study, we selected at random a pool of 724 of their children (using 724 different wells) who were thought to be between 5.75 years and 6.25 years of age, based on original parental intake interviews. For approximately 15 months beginning in December 2003, field staff visited families, on a random basis, at home to verify child age and school attendance, to discuss the proposed study, and to make appointments for clinic visits. To increase the applicability of the assessment of intellectual function to this population, only children who had begun school were included. Of the initial pool of 724 children, the families of 484 were visited at home. Based on the following criteria, 183 children were excluded: 44 families had moved, or else the family or child was traveling outside the region at the time of the visit; 26 families refused participation; 102 families were outside the designated age range; 3 children had died since their families were enrolled; and 8 were excluded for other reasons, such as mother’s illness; ultimately, 301 children were assessed.

### Procedure

Between 2004 and 2005, home visits confirmed the presence of a 6-year-old child in the home, verified the child’s school attendance, and assessed the opportunities for stimulation in the home [using an abridged version of the Home Observation for the Measurement of the Environment (HOME) (Bradley et al. 1989), as discussed below], and established the family’s willingness to participate in the assessment. Children and their mothers came to our field clinic, where children participated in the assessments described below and received a medical examination by a study physician. Weight, height, and head circumference were measured. Children provided urine specimens for the measurement of urinary As and creatinine, and were asked to provide a blood sample for the measurement of blood lead (BPb) and hemoglobin concentrations. Of the 301 children assessed, 143 agreed to provide blood samples. Information on family demographics (e.g., parental age, education, occupation) was available from the baseline interview of parents during their enrollment in the cohort study. Information on the primary source of drinking water was obtained from the child’s mother. Parents were asked whether their home included a television and about child birth order. As an additional surrogate for social class, the type of roofing on the well-owner’s home was recorded as thatched (lowest), tin, or cement (highest).

### Measures

#### Water analyses

Water arsenic concentrations of tube wells at each child’s home were obtained during a survey of all wells in the study region (van Geen et al. 2003b), and shipped to Columbia’s Lamont Doherty Earth Observatory for analysis. Water samples were analyzed by graphite furnace atomic absorption (GFAA), which had a detection limit of 5 μg/L. Those water samples found to have < 5 μg/L were subsequently reanalyzed by inductively coupled plasma–mass spectrometry (ICP-MS), which has a detection limit of 0.1 μg/L (Cheng et al. 2004). All well-water samples were analyzed for Mn by standard flame atomic absorption spectrophotometry.

#### Biochemical measurements

Urinary arsenic concentrations were assayed by GFAA at the Mailman School of Public Health, using a Perkin-Elmer Analyst 600 system as described by Nixon et al. (1991). Our laboratory participates in a quality control program coordinated by P. Weber at the Québec Toxicology Center, Québec, Canada. During the course of this study, intraclass correlation coefficients between our laboratory’s values and samples calibrated at Weber’s laboratory were 0.99. Levels of As in urine were also adjusted for urinary creatinine levels, which were analyzed by a standard colorimetric method based on Jaffe’s reaction (Heinegard and Tiderstrom 1973).

Venous blood samples were obtained for measurements of BPb (Fernandez and Hilligoss 1982) from 143 children. Whole blood samples were appropriately stored and transported to Columbia University, where the laboratory participates in the BPb quality control program of the Centers for Disease Control and Prevention (CDC). Intraclass correlation coefficients between our laboratory’s values and samples calibrated at CDC averaged 0.99. Of 27 potential sociodemographic and exposure covariates, children providing blood samples differed significantly from those declining only in their weight, school attendance, and Mn exposure: Those declining were lighter, had attended school approximately a month longer, and used wells with higher levels of Mn than those providing blood samples (data not shown).

#### School attendance

During the home interview, mothers were questioned about their child’s school attendance. Although only children who were attending school were included, children differed in the number of months since they had begun attending school (attendance usually begins in the January closest to the child’s 6th birthday). Children also differed in the number of days per week (of a maximum of five) that they regularly attended school. Accordingly, we created a continuous measure of school attendance that multiplied the number of months the child had been enrolled in school by the number of days per week that she or he regularly attended and a constant 4.348 for averaged number of weeks per month.

#### Opportunities for stimulation in the home

Research consistently identifies measures of the child-rearing quality of the home as strongly predictive of children’s intellectual function (Bradley et al. 1989). The instrument generally used to assess childrearing qualities is the HOME, which offers a version appropriate to the age of the children studied (Bradley et al. 1989). Of the 59 HOME items, many, including availability of a library card and access to a desk, were culturally inappropriate in the present setting. Accordingly, we selected a limited set of items, supplemented by similar-type items that asked about materials and opportunities available to the child at home, and administered this as a structured interview during the initial home visit. The six items were summed, and the resultant scale had a Cronbach’s alpha of 0.74. The items included presence of a clock in the home; displayed artwork or a current wall calendar; availability in the immediate household family of a watch; presence of any age-appropriate toy; and whether the child had made a trip away from the region in the preceding 6 months.

#### Children’s intellectual function

The Wechsler Preschool and Primary Scale of Intelligence, 3rd edition (WPPSI-III) (Wechsler 2002), suitable for children 2.5 years through 7.25 years of age, consists of 14 subtests that together provide composites, including Full-Scale, Verbal and Performance IQ, Processing Speed Quotient, and General Language Composite. In the age range of the children tested here, seven subtests must be administered; the test manual allows substitution of certain subtests when others are considered inappropriate. Following such guidelines, we derived a test battery that included the following subtests: Block Design, Information, Matrix Reasoning, Comprehension, Picture Completion, Similarities, and Coding. This battery allowed derivation of Composite scores in all domains except for the General Language Composite. The suggested order of administering subscales was followed. Neither the WPPSI-III (Wechsler 2002) nor any other recently well-standardized child IQ test has been adapted or standardized for use in Bangladesh.

In Araihazar, living conditions differ dramatically from those in Western settings where this test was developed, which necessitated adaptations for use in this culture, as described earlier (Wasserman et al. 2004). As in our earlier work, certain items were eliminated or altered slightly for the present application. Eliminated items included one (requiring a stamp on a letter) from the Information subscale, four from Matrix Reasoning (items 6, 8, 11, and 15), one from Similarities (“rain and snow”), and two from Comprehension (need for dogs to have tags, need to save water) that were deemed culturally inappropriate with no recognizable analog; close substitutions were made for four others from the Similarities subscale (“Chocolate and ice cream” for “Cookies and ice cream,” “Mangoes and bananas” for “Apples and oranges,” “Flutes and drums” for “Guitars and drums,” and “Dogs and cows” for “Dogs and cats”). The WPPSI-III subtests include items of graduated difficulty, with more points awarded for harder items or faster completion. As in our earlier work, we summed these weighted items across Verbal, Performance, Processing Speed, and Full-Scale domains to create Verbal, Performance, and Full-Scale raw scores. We transformed these into measures of estimated Verbal, Performance, Processing Speed, and Full-Scale IQ, despite the obvious limitations in application of “IQ” to this population.

Maternal intelligence was assessed with Raven’s Standard Progressive Matrices, a non-verbal test relatively free of cultural influences (Raven et al. 2000).

### Translation and training

All tests and interviews were translated (and back-translated) between Bangla (Bengali) and English. As noted, items deemed to be culturally inappropriate were altered or omitted. Materials were piloted to ensure maternal and child comprehension, and then two testers were trained by a competent tester (G.A.W.), and then continued with supervised practice sessions for 2 weeks. All written test responses were rechecked when data were sent to Columbia for entry. Percent agreement for two testers, calculated from 15 tests, ranged between 0.93 for Comprehension and 1.0 for Block Design; kappas ranged between 0.84 and 1.0.

### Statistical analyses

#### Outcomes

Because of concerns about the application of U.S. standardization of the WPPSI-III to Bangladeshi children, we first conducted analyses that predicted Verbal, Performance, Processing Speed, and Full-Scale raw scores. Because the psychometric properties of IQ scores are more familiar to readers, we also applied the same analytic models to the prediction of estimated Verbal IQ, Performance IQ, Processing Speed Quotient, and Full-Scale IQ.

#### Covariate adjustment

In Bangladesh, grammar school extends to 5th grade. Thus, mother’s and father’s education were categorized as “None,” “1–5 years,” and “6–16” years. Parental occupation was recoded as “Laborer/Farmer,” “Factory/Other paid job,” “Business,” or “Missing/Other.” Because just 7% (*n* = 20) of mothers reported working outside the home, only paternal occupation was considered as a potential control variable. From a pool of potential demographic covariates, we retained those that were empirically or theoretically importantly related to child intelligence, as well as those that made an initial contribution (at significance *p* < 0.20 or better), in initial regression analyses, either to any of the outcomes of interest or to the measures of As exposure. Two measures (availability of electricity within the home, and child’s access to television) were dropped from analyses because they lacked variability (90% positive) and because they contributed minimally to only one or another outcome. Based on our previous finding that water Mn was associated with intellectual function in 10-year-olds, regression models adjusted for water Mn concentrations in children’s household wells.

#### Analytic model

The analyses first sought to predict the outcomes of interest from the set of sociodemographic factors using linear regression models; once this “core” model was derived, we examined the incremental association of As exposure measured continuously and log-transformed. Because the pool of potential sociodemographic factors was larger for the present study (including school attendance and opportunities for stimulation in the home), we derived a new “core” model rather than applying the one used in our earlier study of 10-year-olds (Wasserman et al. 2004). To illustrate dose–response relationships, we repeated our analyses, categorizing children into quartiles based on water As concentration. We next repeated these analyses for the subset of children providing blood samples for the measurement of BPb, measured continuously. In all analyses, to make distributions approximately symmetric, water As was log-transformed and water Mn and school attendance were square-root transformed. For the most part, analyses are based on *n* = 301 children. Analyses adjusting for BPb are based on *n* = 143.

## Results

### Sample characteristics

Table 1 presents descriptive information for all demographic, water and biochemical variables. Average child age was slightly older than 6 years; approximately half the sample was male; most children had regular access to a television. On average, mothers and fathers reported 3.3 and 3.7 years of education, respectively. The average child was 108.5 cm tall, weighed 16 kg, and had a body mass index (BMI) of 13.6, values that correspond to approximately the 5th percentile by U.S. norms (CDC 2003). Of a maximum of 6 items on the scale of Home Stimulation, the average child received a score of 2.7. On average, children attended school 4 days/week, and had been attending for the previous 10 months.

### Exposure characteristics

Water As concentrations ranged from 0.10 to 864 μg/L, with a mean (120.1 μg/L) and distribution comparable to that in the larger set of 6,000 contiguous wells in Araihazar (van Geen et al. 2003b). Concentrations were very similar to those reported for our earlier study of 10-year-olds (117.8 μg/L; Wassserman et al. 2004). The mean water Mn concentration of 1,302 μg/L was well in excess of the U.S. and WHO recommended maximum contaminant level (MCL) of 400 μg/L (WHO 2006), with a range up to 5,549; 88.4% of children were consuming water in excess of the MCL for Mn. The association between water As and water Mn was significant (Spearman *r* = 0.26; *p* < 0 .0001) but not so strong as to preclude examination of their independent effects on child intelligence. The Spearman correlation (necessitated by skewed distributions) between water As and urinary As (*r* = 0.31, *p* < 0.0001) was moderate but lower than the value we previously reported for 10-year-olds (*r* = 0.45, *p* < 0.0001). In the subsample of children for whom BPb measures were obtained (*n* = 143), correlations between BPb and well water As (−0.19) or urinary As (0.01) were not significant.

### Relationship between covariates and intellectual function

Linear regression analyses predicting test raw scores from the socio-demographic features retained in the final “core” model (with and without water Mn) revealed generally better scores in children of more educated mothers and of mothers with higher Raven scores, those living in more stimulating households, those who attended school more often, and those who were taller and had a larger head circumference (data not shown).

### Relationship between well water As and intellectual function

Table 2 presents associations between water As and intellectual function, before and after adjustment for water Mn and for sociodemographic features. Before adjustment, water As was negatively but not significantly associated with all outcomes. In unadjusted analyses, water As explained 0.21%, 0.09%, 0.001%, and 0.07% of the variance in Performance, Processing Speed, and Full-Scale raw scores, respectively and a negligible proportion (< 0.001%) of the variance in Verbal raw scores. After covariate adjustment, water As showed significant but smaller negative associations with Performance raw scores (B = −0.48, *p* < .05), explaining 1.04% of the variance. After covariate adjustment, the association between water As and Full-Scale raw scores was marginally significant (B = −1.06, *p* < 0.07), explaining 0.72% of the variance in that outcome; water As contributed significantly to Processing Speed raw scores (B = −0.54, *p* < 0.05), explaining 0.81% of the variance in that measure. Associations between water As and Verbal raw scores did not approach significance. Water Mn made no contribution to any of the intellectual function outcomes. Water As made no contribution to IQ outcomes (data not shown).

### Dose–response relationships between water As and intellectual function

We next examined the adjusted Full-Scale, Performance, Verbal, and Processing Speed raw scores by As categories defined as quartiles (0.1–20.9, 21–77.9, 78–184.9, 185–864). As water As increased, there were dose-dependent changes in adjusted and unadjusted scores (data not shown). With adjustment, compared with the lowest category of As exposure, those in the second and third categories received significantly lower Performance raw scores (B = −2.8 and −2.4; *p* < 0.03 and *p* = 0.05, respectively for the second and third categories). With adjustment, compared with the lowest category of As exposure, those in the fourth category (with the highest exposure) had marginally significantly lower Full-Scale and Processing Speed raw scores (B = −5.70, *p* < 0.06 and B = −2.48, *p* < 0.09, respectively). Verbal raw scores did not differ significantly across categories of exposure.

### Relationship between urinary As and intellectual function

We next examined relationships between total urinary As concentration, as micrograms per gram creatinine, and child intellectual function. After adjustment for core variables, the associations between urinary As and measures of intellectual function were not statistically significant for Full-Scale (B = −1.78, *p* = 0.21), Performance (B = −0.81, *p* = 0.17), Verbal (B = −0.16, *p* = 0.81), or Processing Speed raw scores (B = −0.93, *p* = 0.18), but were in the anticipated direction.

### Relationship between BPb and intellectual function

In the subset of 143 children with BPb measures, no associations were found between BPb and any outcome, regardless of control for water As. The associations between water As and outcome were not altered by the addition of BPb in the model (data not shown).

## Discussion

This article represents our second systematic study of the effects of As on children’s intellectual function, with findings that essentially corroborate our earlier results with 10-year-old children in Bangladesh (Wasserman et al. 2004). Exposure to As from drinking water is associated with reduced scores on measures of intellectual function, before and after adjusting for water Mn, for BPb, and for socio-demographic features known to contribute to intellectual function. With covariate adjustment, water As remains significantly negatively associated with both Performance and Processing Speed raw scores, and associations for Full-Scale raw scores approach statistical significance; Verbal scores were unaffected.

Other investigators have also observed adverse associations between As exposure and children’s intellectual function. After controlling for demographic covariates, a negative association between urinary As and verbal intelligence was reported among 80 children living near a lead smelter in Mexico (Calderon et al. 2001). A recent small pilot study of 31 children, 11–13 years of age, residing in a former lead and zinc mining site containing tons of mining waste, or chat (Wright et al. 2006), found adverse associations between both hair As and hair Mn, and general intelligence scores, particularly verbal scores. In both age groups studied in Bangladesh—i.e., the present sample and our earlier report with 10-year-olds (Wasserman et al. 2004)—we did not find an association between As exposure and aspects of verbal intelligence. Our results are more in keeping with those of an ecologic study in Taiwan (Tsai et al. 2003) that compared adolescents from regions with and without elevated As in well water. After control for sociodemographic factors, Taiwanese adolescents in the exposed group showed inconsistently poorer scores on Performance-type tests; relative to those without exposure, some outcomes were adversely affected in adolescents with low (but not high) exposure.

The pattern seen here is very similar to that previously observed in older children. However, for 6-year-olds, the magnitude of the association between water As and intellectual function is weaker, and the dose–response relationship is less stable. The present findings, then, might indicate that younger children, with their shorter exposure, might manifest less adverse consequences than those seen in older, longer-exposed children. Before expressing cautious optimism at these findings, we should first explore several alternative possible explanations for these apparent differences.

First, there have been considerable mitigation activities since the original well survey in 2000. Between January and June of 2001, we labeled 5,000 of the 6,000 wells in our study region with the As test results and a picture of either a skull and cross-bones (for wells ≥ 50 μg/L As) or a picture of a child drinking a glass of water (for those < 50 μg/L As). The remaining 1,000 well owners were informed orally and given health cards with the test results. In addition, we carried out health education programs in many villages in our study area to encourage well switching. From early 2001 to 2004—that is, when the fieldwork for the current study began—these interventions led many families to gradually shift their water consumption from a well high in As to one with less As (Opar et al. 2007; van Geen et al. 2002). This shift would have had considerably less impact on children from our earlier study (Wasserman et al. 2004), who were assessed in 2002. Among adults in this region, urinary As concentrations fell by 25% among those who had wells with > 50 μg/L (data not shown). Thus, in the current study, it is possible that our mitigation efforts may have led to some exposure misclassification based on baseline water As; this would bias the effect toward the null. Although the mean urinary As concentration of children in the current study (348 μg/g creatinine) was very similar to that in our previous study of 10-year-olds (297 μg/g creatinine), the correlation between water As and urinary As adjusted for creatinine concentration is lower for the present 6-year-olds than that seen with earlier 10-year-olds (*r* = 0.42 and 0.56, respectively, *p* < 0.04). Similar differences appear for associations between water As and urinary As, unadjusted for creatinine concentration (*r* = 0.31 and 0.42, respectively, *p* < 0.06). If well characteristics (ascertained a few years before assessment of both urinary As and intellectual function) map less closely onto urinary As concentration in the present study, this would suggest that some well switching in the years between measurement of well water and of intellectual function has, indeed, taken place.

A second possible explanation for the different effect sizes observed across the two age groups relates to the lower stability of estimates of child intelligence at younger age (e.g., Bartels et al. 2002; Petrill et al. 2004). Test scores for younger children may be more unreliable, although we have increasing confidence that scores after 6 years of age are really assessing “intelligence.” Also, the standard deviations for the various measures of intellectual function show very similar standard deviations in our samples at 6 and 10 years of age, so that it does not appear that there is substantially more measurement “noise” in the younger sample.

It seems, then, that children in the present study had less lengthy exposure to As, and that their reduced exposure may have attenuated, to a degree, the adverse consequences for intellectual function. If further study confirms the reversibility of adverse consequences after well mitigation, this might mean that well interventions delivered to very young children could have a significant impact on reducing negative impact on intellectual function.

### Mn and Pb exposure

The literature concerning adverse effects of Mn on child intelligence is growing, though we failed to detect any adverse association in the current study. However, the current study was not designed to examine effects of Mn exposure on intellectual function; 88.4% of the children were exposed to high water Mn, limiting the power to detect the association. In a study of 10-year-old Bangladeshi children exposed to high levels of Mn (mean = 793 μg/L) and low levels of As (mean = 3 μg/L), water Mn was associated with reduced Full-Scale, Performance, and Verbal raw scores, in a dose–response fashion; the low level of As was not associated with outcome (Wasserman et al. 2006). Two earlier ecologic studies from China found similar evidence. Ninety-two pairs of children 11–13 years of age, matched for age, sex, grade, family income, and parental education, from regions with and without high levels of Mn in sewage irrigation and drinking water, were assessed using the WHO (WHO 1986) neurobehavioral core test battery (Peng et al. 1994). Hair Mn levels were significantly higher in children in the exposed village. On the neurobehavioral exams, children in the exposed village scored lower in digit span, Santa Ana manual dexterity of the nonpreferred hand, digit symbols, and Benton visual retention. In addition, among children in the exposed area, regression analyses also revealed adverse associations with those outcomes, though no attempt was made to adjust for covariates. A follow up study of the same children reported significantly lower serum levels of four neurotransmitters, including dopamine, norepinephrine (NE), acetylcholine (AchE), and 5-hyroxytryptamine (5-HT) (Zhang et al. 1995). Exposure was related both to serum levels of 5-HT, NE, and AchE and to overall examination scores.

As in our previous study, we did not observe the anticipated relationship between BPb and child intellectual function. We had minimal statistical power to do so, however, because approximately half of the study children refused to provide a blood sample.

### Limitations

As in our earlier work, we cannot comfortably make a statement about IQ points lost in relationship to As exposure, because it is impossible to apply the U.S. standardization norms to the generation of IQ scores in the current study population. Although the procedures we have followed for adapting a standardized instrument for use in very different cultural setting are sound, our assessment likely measures an analogue of IQ, and not IQ itself.

In summary, this second cross-sectional study of As exposure expands our concerns about As neurotoxicity to a younger age group. Clearly additional systematic research is needed to fully define the dose–response relationships and vulnerable periods of brain development.

## Figures and Tables

**Table 1:** Sample characteristics [no.^a^ (%) or mean ± SD].

Variable	Value
Male sex	150 (49.8)
Television access	259 (86.1)
Electricity in home	270 (89.7)
House type	
Thatched roof or poorer	41 (13.6)
Corrugated tin	235 (78.1)
Concrete construction	25 (8.3)
Father’s occupation	
Other/missing	52 (15.3)
Laborer/farmer	55 (18.3)
Factory/other paid job	107 (35.6)
Business	93 (31.0)
Child age (years)	6.1 ± 0.18
School attendance (days/week)	4.3 ± 1.6
Months attending school	10.3 ± 4.2
Full-Scale “IQ” (*n* = 296)	75.9 ± 7.4
Verbal “IQ” (*n* = 299)	83.0 ± 7.3
Performance “IQ” (*n* = 298)	73.5 ± 7.9
Full-Scale Raw Score (*n* = 296)	99.6 ± 21.8
Verbal Raw Score (*n* = 300)	39.3 ± 9.4
Performance Raw Score (*n* = 298)	42.6 ± 8.1
Processing Speed Raw Score (*n* = 299)	17.1 ± 10.4
Height (cm)	108.5 ± 5.6
Weight (kg)	16.0 ± 2.0
BMI	13.6 ± 1.1
Head circumference (cm)	48.4 ± 1.5
Mother’s education (years)	3.3 ± 3.5
Father’s education (years; *n* = 295)	3.7 ± 4.0
Mother’s age (years)	30.0 ± 5.7
Father’s age (years)	38.5 ± 6.9
Mother’s Raven score (*n* = 297)	14.2 ± 3.4
Home Stimulation	2.7 ± 1.8
Water Mn (μg/L)	1,302 ± 821
Water As (μg/L)	120.1 ± 134.4
Urinary As (μg/L)	110.7 ± 132.8
Urinary creatinine (mg/dL)	36.6 ± 26.8
Urinary As (μg/g creatinine)	347.7 ± 352.7
BPb (μg/dL)	11.9 ± 3.5

a Except where noted, sample size is 301.

**Table 2:** Regression coefficients relating Water As, before and after covariate adjustment to Verbal, Processing Speed, and Full-Scale raw scores.

Measure	Performance raw score B (SE)	Verbal raw score B (SE)	Processing Speed raw score B (SE)	Full-Scale raw score B (SE)
Before adjustment				
Water As	−0.19 (0.25)	−0.13 (0.29)	−0.17 (0.32)	−0.30 (0.67)
After adjustment				
Maternal education (years)				
None (reference)				
1–5 years	0.61 (1.02)	−0.69 (1.21)	1.97 (1.20)	1.51 (2.44)
6–15 years	2.79 (1.25)**	0.13 (1.48)	2.49 (1.46)	5.57 (2.98)*
Maternal intelligence	0.09 (0.13)	0.09 (0.16)	0.43 (0.16)#	0.62 (0.32)**
Home Stimulation	0.97 (0.26)##	1.53 (0.31)##	1.08 (0.30)##	3.45 (0.61)##
School attendance	0.38 (0.10)##	0.31 (0.12)**	0.87 (0.12)##	1.55 (0.24)##
Height	0.28 (0.08)##	0.33 (0.10)#	0.37 (0.10)##	0.90 (0.20)##
Head circumference	0.61 (0.29)**	0.62 (0.35)	0.49 (0.35)	1.81 (0.71)**
Water manganese	0.02 (0.04)	0.02 (0.05)	0.02 (0.04)	0.05 (0.09)
Water arsenic	−0.48 (0.24)**	−0.18 (0.28)	−0.54 (0.28)**	−1.06 (0.57)*
Total *R*^2^	27.9%	21.1%	38.3%	42.4%

Abbreviation: B, unstandardized regression coefficient; *R*^2^, sum of variance explained.

* *p* < 0.07;

** *p* ≤ 0.05;

# *p* < 0.01;

## *p* < 0.001.
